# HEPNET Position Statement-I, Case Definition, Classification, Screening & Diagnosis of Metabolic Dysfunction-Associated Steatotic Liver Disease (MASLD) in Pakistan: A Resource for Primary and Secondary Care Physicians

**DOI:** 10.12669/pjms.41.3.10081

**Published:** 2025-03

**Authors:** Bushra Ali, Lubna Kamani, Adnan Salim, Altaf Alam, Bader Faiyaz Zuberi, Javed Iqbal Farooqi, Altaf Baqir Naqvi, Zeeshan Ali, Shahid Majid, Zahid Yasin Hashmi, Asad A Choudhry, Muhammad Salih, Anwar Ahmed Khan, Syed M. Zahid Azam, Zaigham Abbas, Masood Siddique, Arif Amir Nawaz

**Affiliations:** 1 Bushra Ali, Fatima Memorial Hospital College of Medicine and Dentistry, Lahore, Pakistan; 2 Lubna Kamani, Liaquat National Hospital, National Medical Center, Karachi, Pakistan; 3 Adnan Salim, Shaikh Zayed Medical Complex Lahore, Pakistan; 4 Altaf Alam, Consultant Gastroenterologist, Evercare Hospital Lahore, Lahore, Pakistan; 5 Bader Faiyaz Zuberi, OMI Hospital Karachi, Pakistan; 6 Javed Iqbal Farooqi, Lifecare Hospital, Hayatabad Peshawar, Pakistan; 7 Altaf Baqir Naqvi, Medicare Hospital Multan, Pakistan; 8 Zeeshan Ali, Jinnah Sindh Medical University & Jinnah Postgraduate Medical Center Karachi, Pakistan; 9 Shahid Majid, The Indus Hospital and Health Network, Karachi, Pakistan; 10 Zahid Yasin Hashmi, Liver Center, Faisalabad, Pakistan; 11 Asad A Choudhry, Consultant Gastroenterologist, Chaudhry Hospital, Gujranwala; 12 Muhammad Salih, Shifa International Hospital, Islamabad, Pakistan; 13 Anwaar Ahmed Khan, Doctors Hospital and Medical Center, Lahore, Pakistan; 14 Syed M. Zahid Azam, National Institute of Liver & GI Diseases, Dow University, Karachi, Pakistan; 15 Zaigham Abbas, Ziauddin University Hospital Clifton Karachi, Pakistan; 16 Masood Siddique, Army Medical Core, Rawalpindi, Pakistan; 17 Arif Amir Nawaz, Fatima Memorial Hospital, Lahore, Pakistan

**Keywords:** Metabolic dysfunction-associated fatty liver disease, metabolic dysfunction-associated steatotic liver disease

## Abstract

The Hep-Net position paper comes at a significant time in the history of Metabolically Associated Fatty Liver Disease (MAFLD) due to the rapid rise in this disease entity in the past decade. Metabolically Associated Fatty Liver Disease, by its very name, encompasses several common metabolic disease entities, top among those being diabetes and obesity. For Pakistan, the situation is serious as it is among the top 10 countries globally regarding the prevalence of obesity and number one in terms of diabetes, with over a quarter of adults affected. There remains slight ambiguity as regards the nomenclature of MAFLD, with western societies preferring to remove the word “fatty” and substitute with `’steatotic” i.e. MASLD.

Regardless of names/titles the metabolic nature of the disease and its management remains the same and fortunately, that is something where universal consensus is present. Under the umbrella of Hep-Net, eminent hepatologists from all over Pakistan have pooled their efforts to formulate guidelines that are specifically tailored to the Pakistani population, its specific lifestyle and relevant interventions that are needed to treat fatty/steatotic liver disease. By virtue of its multi-systemic consequences, metabolic fatty liver disease represents the most significant and expensive disease entity, globally. Prevention, through public education and timely intervention in diagnosed cases will serve to avert a healthcare storm that will far outweigh viral hepatitis.

## INTRODUCTION

Metabolic dysfunction-associated fatty liver disease (MAFLD) and metabolic dysfunction-associated steatotic liver disease (MASLD) are new names for liver diseases that are linked to Hepatic Steatosis (HS). This disease was previously known as non-alcoholic fatty liver disease (NAFLD).^1^-^3^ Instead of exclusion, MAFLD/MASLD focuses on the metabolic syndrome’s role in pathogenesis & progression of the disease, enabling it to be a more accurate and positive diagnostic criteria.^4^

The new label MASLD is intended to destigmatize the illness by eliminating the negative connotation of “non-alcoholic” and “Fatty” to identify the people with severe liver disease and poor clinical outcomes more effectively^5^, while using the terms “fatty” and “alcoholic” was not only stigmatizing for some patients^3^ this may be perceived as judgmental. The metabolic factors, rather than alcohol consumption, are the primary drivers of this condition^5^-^7^ and both can also coexist as well. This change reflects a more comprehensive understanding of the pathophysiology of the condition. The new term not only emphasizes the central role of metabolic factors, such as obesity, insulin resistance, hypertension, dyslipidemia and type 2 diabetes mellitus, in the development and progression of the disease,^2^,^4^,^5^ it also allows for coexistence of different liver disorders like alcoholic liver disease, DILI, HCV or cryptogenic steatosis, which is more realistic as patients may have more than one etiology. The major international societies as well as the Pakistan Society of Hepatology in its recent position statement has endorsed it.^1^ The pathophysiology of MASLD involves hepatic lipid accumulation and mitochondrial dysfunction, with differential methylation within mitochondrial DNA being implicated in disease progression.^8^-^14^

NAFLD/MASLD have rising global prevalence reaching up to one third of its population, it is increasing continuously at alarming rate, requiring urgent and comprehensive strategies to raise awareness and address all aspects of the disease on local, regional, and global levels.^15^-^17^ According to projections, by the year 2040, more than 50% of adults are expected to develop MASLD/NAFLD. Greater efforts are required to enhance public awareness of MASLD and to identify sustainable remedies that target the primary causes of the illness.^18^ MASLD/NAFLD is increasingly prevalent in Pakistan, driven by rising obesity rates, unhealthy lifestyles, and poor health awareness. Studies indicate that NAFLD affects about 15% of the general population, with a higher prevalence among type-2 diabetic patients, where it can reach up to 77.5%.^19^-^22^ The prevalence & progression of MASLD is notably high among populations with obesity (central or visceral), type 2 diabetes, hypertension, genetic factors, sedentary lifestyle, dyslipidemia and poor dietary habits.^2^,^20^,^23^-^27^

## RATIONALE

Diabetes and obesity are twin epidemic with a massive burden and escalating disease prevalence in Pakistan.^28^-^32^ As diabetes and obesity are the main drivers of metabolic syndrome and MASLD,^33^ it is expected that we will have to face a very high burden of MASLD, though our national prevalence data is non-existent. A locally perceived and regionally customized guidance document with a multipronged approach is the urgent need of the time, not only to adopt the new nomenclature, but also for early detection and diagnosis of MASLD, an agreed management plan to target the main driver of the disease process and its associated risk factors and more so to identify which patient need a referral to specialist center. This should target not only hepatologist but also internists, endocrinologists, cardiologists, primary care & secondary care physicians.

## METHODS

HEPNET Pakistan is a group of hepatologist working together to improve the liver health in Pakistan. Keeping the complexity of diagnosis, changing nomenclature & rising prevalence of MASLD, HEPNET decided to draft this position statement, multiple in-person & virtual meetings were convened to reach an agreement & develop this guidance statement for Pakistan. Due to lack of in-depth local data regarding epidemiology, risk factors and management on MASLD it was decided to write a position statement and the modified Nominal Group Technique (mNGT) was used to reach an agreement. PubMed & google scholars were used to search local and international published data. Search was targeted to search terms like NAFLD, NASH, MASLD, MAFLD for epidemiology, risk stratification, natural history, risk stratification, diagnosis, non-invasive test, liver fibrosis, HCC, lifestyle modifications, weight loss therapies & management were used to review the data. Sub-groups were formed and assigned to the different sections to review data and formulate recommendations. All recommendations were compiled with-in the sub-groups first, and then all members were invited for voting, strength of each recommendation was drawn from member voting and following grading was used to rate the recommendations.

### Who should Be the target:

This should target hepatologists, gastroenterologists, internists, endocrinologists, cardiologists, primary care & secondary care physicians.

### Steatotic Liver Disease (SLD):

Fatty liver or HS is accumulation of fat in hepatocytes. It may be caused by a variety of factors, both metabolic and non-metabolic^34^ so Steatotic Liver Disease (SLD)is a newly proposed umbrella term. MASLD is often the primary cause, linked to metabolic syndrome, which collectively increases the risk of HS and its progression to more severe liver conditions such as steatohepatitis, fibrosis, cirrhosis & HCC^35^-^37^ but there are other causes also. Excessive alcohol consumption also leads to fat accumulation in the liver cells. In addition, some medications, including cytotoxic or chemotherapeutic agents, can cause HS.^38^,^39^

In mice model, genetic factors leading to lack of miR-122a, which affects triglyceride transfer protein expression, were found to contribute to the development of steatosis.^40^,^41^ Additionally, an increase of oxidative stress and abnormalities in proteins such as Hsp70.1 and BHMT, which play roles in the metabolism of fatty acids and choline respectively, might result in the buildup of fat in the liver.^42^,^43^ It has been observed that alterations in bile acid metabolism after cholecystectomy have also contributed to HS.^44^ Poor eating habits and a sedentary lifestyle are also significant contributors.^45^-^47^ Based on histological appearance conventionally HS has been traditionally classified as macro or micro vesicular, both having its own etiologies as under,

**Table:** 

HEPNET Recommendation		
Strength	Level	Modified Nominal Group Technique (mNGT) Criteria
Strong	I	More than 75% of the group members agreed
Moderate	II	More than 50% of the group members agreed but less than 75%
Week	III	More than 25% of the group members agreed but less than 50%
None	N	Less than 25% of the group members agree

### Macrovesicular Steatosis(2):

Drinking too much alcohol, HCV GT3, Wilson disease, lipodystrophy, starvation, parental nutrition, elevated lipoproteinemia, medications (amiodarone, lomitapide, methotrexate, tamoxifen, corticosteroids, mipomersen)

### Microvesicular Steatosis(2):

Acute fatty liver of pregnancy, HELLP syndrome, retinopathy, valproic acid and antiretroviral drugs, inborn errors of metabolism (lecithin cholesterol acyltransferase deficiency, cholesterol ester storage disease, Wolman’s disease).

HEPNET RECOMMENDATION# 1 & 2

1. HEPNET recommends nomenclature of Metabolic Dysfunction Associated Steatotic Liver Disease (MASLD) should be adopted to replace existing term NAFLD in Pakistan. (I)

2. Diagnosis of Steatotic Liver Disease (SLD) should be established based on any imaging evidence of HS (ultrasound, transient elastography, CT scan or MRI). (I)

## CLINICAL EVALUATION OF SLD

The clinical evaluation of SLD relies on a combination of clinical assessment, laboratory tests, and imaging modalities to provide a comprehensive understanding of the disease, guide management, and monitor progression.

### Patient History:

A detailed medical history to identify various risk factors and potential etiologies of HS is essential in all cases. Key aspects to cover include:

- ***Alcohol Consumption:*** Detailed assessment of alcohol intake to determine effect of alcohol over MASLD. The CAGE questionnaire is quick and easy to administer, making it ideal for initial screening, particularly in settings where time is limited. However, it may miss hazardous drinking that has not yet resulted in significant harm. On the other hand, the AUDIT is more comprehensive, capturing a wider spectrum of alcohol use disorders, including hazardous drinking that could benefit from early intervention.^48^
- ***Metabolic Factors:*** History of obesity, type 2 diabetes, dyslipidemia, and metabolic syndrome.
- ***Medications:*** Use of medications known to cause fatty liver, such as corticosteroids, methotrexate, and tamoxifen.
- ***Diet and Lifestyle:*** Dietary habits, physical activity level, and rapid weight loss or gain.
- ***Family History:*** Family history of liver disease, viral hepatitis, diabetes, or other metabolic disorders.

### Physical Examination:

A comprehensive physical examination focuses on identifying signs of liver disease and associated conditions:

- ***General Appearance:*** Specially observe the blood pressure, height/ weight for BMI & waist circumference (WC) for central adiposity.
- ***Abdominal Examination:*** Palpation for hepatomegaly (enlarged liver), which may indicate fatty liver.
- ***Signs of Liver Disease:*** Checking for signs such as jaundice, ascites, spider angiomas, and palmar erythema, which suggest advanced liver disease or cirrhosis.
- ***Other Signs:*** Examination for signs of insulin resistance, such as acanthosis nigricans.

### Laboratory Workup:

- Blood Tests: Laboratory tests are crucial for assessing liver function, metabolic status, and ruling out other causes of liver disease:
- ***Liver Enzymes/ Liver Function:*** Elevated levels of alanine aminotransferase (ALT) and aspartate aminotransferase (AST) are common in hepatic steatosis but are not specific. Bilirubin, alkaline phosphatase (ALP), gamma-glutamyl transferase (GGT), and albumin levels to evaluate overall liver function.
- ***Lipid Profile:*** Assessment of cholesterol, triglycerides, and HDL/LDL levels to evaluate dyslipidemia.
- ***Glucose Metabolism:*** Fasting glucose, insulin levels, and HbA1c to assess for diabetes and insulin resistance.
- ***Viral Serology:*** Tests for viral hepatitis (HBV, HCV), autoimmune markers, and iron studies to rule out other liver diseases.
- ***Complete Blood Count***: For Platelet count which will be essential for calculating APRI & FIB-4 score to assess Liver fibrosis.

HEPNET RECOMMENDATION# 3

3. All patients with SLD on imaging should undergo focused clinical and laboratory evaluations for metabolic risk factors, alcohol intake (AUDIT-C or CAGE questioner), liver biochemistry & viral serology.

HEPNET RECOMMENDATION# 4

4. NIT can be used in patients with SLD to diagnose, evaluate its severity & monitor the disease progression. (II)

### Noninvasive Tests:

Non-invasive tests (NITs) are not only useful in establishing the diagnosis of HS but also stage liver fibrosis and monitor progression of HS & fibrosis. Patients with advanced fibrosis due to MASLD/MASH are susceptible to hepatic decompensation, liver-related mortality & hepatocellular carcinoma.^49^

### Noninvasive Blood Tests for Liver Steatosis and Fibrosis:

Value of NIT is quite established and are now considered essential tools for assessing liver fibrosis, but they are also crucial for establishing the HS, offering a safer and more convenient alternative to liver biopsy. Recent guidelines and research emphasize their diagnostic accuracy and utility in clinical practice.

### Noninvasive Blood Tests for Liver Steatosis: Fatty Liver Index (FLI):

FLI predicts the presence of HS. It combines BMI, WC, triglycerides, and GGT. It is widely used with an AUROC of 0.81 in diagnosing HS in diverse populations.^50^,^51^ Optimal cut-off values vary by study population, race, age and gender. Overall cut-offs of 26.2, for male 30.4 and for female 20.7 are the suggested values.^52^-^54^

### SteatoTest:

It quantifies HS, and its components includes biochemical markers such as ALT, AST, total cholesterol, triglycerides, glucose, and haptoglobin. As test of haptoglobin in not available freely its utility in clinical practice remains limited to clinical trials. Though it has good diagnostic performance for detecting liver steatosis with an area under the receiver operating characteristic curve (AUROC) of 0.81.^55^ For diagnosing grade 2–4 steatosis, A SteatoTest value of 0.30 is highly sensitive for diagnosing moderate to severe steatosis, while a value of 0.70 is highly specific.^56^

### Noninvasive Blood Tests for Liver Fibrosis:

### Aspartate Aminotransferase to Platelet Ratio Index (APRI):

APRI is the most widely used test due to its low cost & shows good performance for advanced fibrosis detection with an AUROC of 0.74.^57^,^58^ It evaluates liver fibrosis from AST levels and platelet count. APRI threshold values for detecting significant fibrosis, advanced fibrosis, and cirrhosis are 0.5, 1.0, and 1.5 respectively.^2^,^59^

### FIB-4 Index:

It combines age, AST, ALT, and platelet count & is reliable for diagnosing advanced fibrosis with an AUROC of approximately 0.77. A low cut-off of 1.3 effectively excludes advanced fibrosis, while a high cut-off of 2.67 for confirms significant fibrosis in MASLD.

### NAFLD Fibrosis Score (NFS):

Identifies the likelihood of advanced fibrosis in NAFLD patients. It uses age, BMI, hyperglycemia, platelet count, albumin, and AST/ALT ratio. It rules out advanced fibrosis in primary care settings, with an AUROC of 0.84.^2^,^60^-^62^

### FibroTest (FibroSure):

Assesses liver fibrosis & effective in diagnosing significant fibrosis with an AUROC around 0.85.^63^-^65^ It measures serum markers such as alpha-2-macroglobulin, haptoglobin, apolipoprotein A1, GGT, and total bilirubin. It’s expensive and not freely available in Pakistan.

### Imaging Techniques:

Noninvasive imaging is critical for diagnosing and quantifying HS. SLD can be detected through various imaging modalities like ultrasound, CT scan, MRI & Liver elastography. Measuring the HS is essential for not only determining the severity of liver disease, but also for monitoring disease progression, and evaluating treatment efficacy. Accurate measurement is also required in research on the natural history and therapeutic interventions in SLD.

### Ultrasound:

Ultrasound is the most used imaging technique for detecting HS as it is non-invasive, widely available and cost-effective. The sensitivity of ultrasound ranges from 60-94%, and its specificity ranges from 66-95% for moderate to severe steatosis.^66^-^68^ However, its accuracy can be influenced by the operator’s experience, patient’s body habitus and equipment being used.^69^-^71^

### Transient Elastography:

Vibration-controlled transient elastography (Fibro Scan) or Shear-wave elastography (SWE) are primarily used to assess liver fibrosis,^72^-^74^ it can also quantify HS through controlled attenuation parameter (CAP) or ultrasound attenuated imaging (ATI) measurements respectively. CAP offers a dependable and non-invasive method for identifying and measuring liver fat. It has been shown to have sensitivity and specificity values ranging from 82% to 88% and 82% to 89%, respectively.^75^-^77^ This modality is quick and easy to perform but its major limitation is it may be less accurate in obese or cirrhotic patients.^78^ Optimal cut-off, dB/m 248 (237 to 261), 268 (257 to 284) & 280 (268 to 294) were found useful to differentiate between S0, S1/2 or S3 steatosis with AUROC) 0.88.^62^,^77^,^78^ ATI and SWE have high diagnostic accuracy for detecting different grades of steatosis and fibrosis, with ATI showing AUROC values of 0.93, 0.88, and 0.83 for steatosis grades S1, S2, and S3, respectively.^79^

HEPNET RECOMMENDATION# 5-8

5. FLI is recommended to confirm, quantify & monitor HS.

6. APRI, FIB-4 or NFS are recommended to confirm, quantify & monitor liver fibrosis.

7. Fibro scan & SWE are recommended to confirm, quantify & monitor liver fibrosis.

8. Controlled attenuated parameters measured by either VCTE or SWE are effective in diagnosing SLD, quantify & monitor its progression

### Computed Tomography (CT):

CT scans technique identifies steatosis by measuring liver attenuation values and provides moderate sensitivity (82-93%) and specificity (85-93%) in detecting hepatic steatosis.^80^-^82^ Due to the exposure to ionizing radiation CT remains less favorable for diagnosis & regular monitoring of hepatic fat content. CT can be valuable in cases where additional abdominal pathology is suspected and requires comprehensive abdominal imaging.

HEPNET RECOMMENDATION# 9

9. Liver Biopsy should only be restricted in therapeutic drug trails or where alternate diagnosis is being sought.

### Magnetic Resonance Imaging (MRI):

MRI proton density fat fraction (PDFF) techniques is currently considered one of the most accurate non-invasive methods for diagnosing & quantifying liver fat, with sensitivity and specificity often exceeding 90%.^83^,^84^ MRI is suitable for longitudinal studies as it provides excellent soft tissue contrast without exposure of ionizing radiation, but it is more expensive and less accessible than ultrasound and CT.^68^,^72^,^73^ Currently it’s not available in Pakistan. Magnetic Resonance Spectroscopy (MRS) is a specialized software and needs expertise.^85^-^87^ It measures the magnetic resonance signals of specific lipid protons and is considered the gold standard for non-invasive liver fat quantification. Its clinical use is limited because of high cost, limited availability & lengthy acquisition times.

### Invasive Tests:

### Liver Biopsy:

It has been long considered as the gold standard for diagnosing, staging & monitoring liver disease, especially in ambiguous cases when alternate diagnosis is being considered or when noninvasive tests are inconclusive. But is has its own limitations, firstly, it is an invasive procedure, carrying risks such as pain, bleeding, and infection.^2^,^88^ Moreover, it samples only a small portion of the liver, which may not accurately represent the entire organ’s pathology. The interpretation of biopsy samples can also be subjective, with significant inter- and intra-observer variability among pathologists, potentially affecting the consistency and reliability of the diagnosis.^63^ Artificial Intelligence as Diagnostic Tool. In recent years there have been significant work being undertaken and reported for Ai as diagnostic tool for SLD.^89^ Different modalities alone or in combinations are being tested. To date it’s quite premature to make recommendations but in future it seems promising.

## CASE DEFINITION & DIAGNOSIS OF MASLD AND/OR MASH

**Fig.1:**
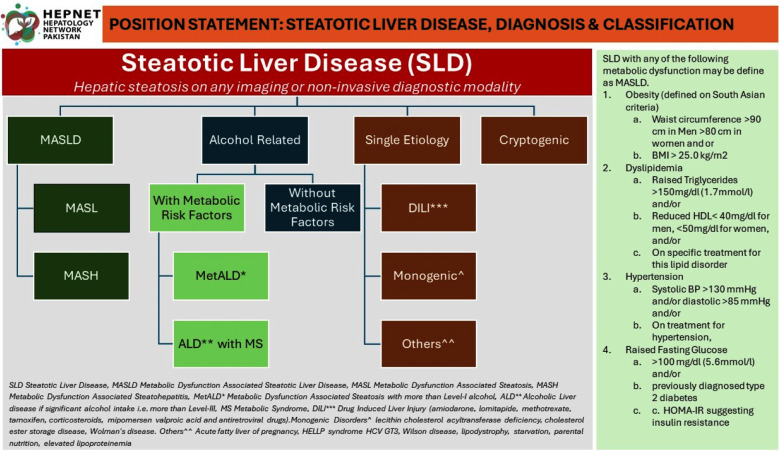
Classification of Steatotic Liver Disease (SLD).

SLD is proposed overarching term for HS seen on any imaging, it will further be classified in either of four groups as shown in Fig.1

### MASLD:

SLD with any of the following metabolic dysfunction, adopted IDF criteria to define Diabetes and MS^90^ or obesity based on South Asian cut offs,^91^,^92^ may be define as MASLD.

1. Overweight or Obesity (defined on South Asian criteria)
  - WC >90 cm in Men >80 cm in women and or
  - BMI ≥ 23.0 kg/m2
2. Dyslipidemia
  - Raised Triglycerides >150mg/dl (1.7mmol/l) and/or
  - Reduced HDL< 40mg/dl for men, <50mg/dl for women, and/or
  - On specific treatment for this lipid disorder
3. Hypertension
  - Systolic BP >130 mmHg and/or diastolic >85 mmHg and/or
  - On treatment for hypertension,
4. Raised Fasting Glucose or Insulin resistance
  - Prediabetes or FBS >100 mg/dl (5.6mmol/l) and/or
  - previously diagnosed type 2 diabetes
  - HOMA-IR suggesting insulin resistance

HEPNET RECOMMENDATION# 10 & 11

10. MASLD should be diagnosed based on if there are any single associated metabolic risk factors is present namely Pre-diabetes, Diabetes, Hypertension, Obesity (define by Asian cut-offs of BMI or Central Obesity) or Dyslipidemia along with imaging evidence HS.

11. Insulin resistance (HOMA-IR) should be checked in patients without diabetes

### MASH:

Patients diagnosed as MASLD when present with significant fibrosis and /or deranged ALT more than upper normal limit (30IU for males & 20IU for females) more than two occasions may be diagnosed as MASH.

## ROLE OF ALCOHOL LIVER DISEASE (ALD) & OTHER ASSOCIATED LIVER DISORDERS AND SLD.

MASLD patients with ALD are a significant and important population, with new nomenclature both can coexist, and it needs further research on natural history, prognosis, and treatment responsiveness to this dual pathology. A detailed history for current & lifetime alcohol consumption will help in assessment & diagnosis of dual etiology in patents with HS. Lately, there has been concerns regarding the so-called “safe limits” for alcohol intake in patients with MASLD,^93^-^95^ even consuming little amounts of alcohol can contribute to a higher risk of progressing to advance cirrhosis and HCC, and it may also result in poor response rates to therapeutic intervention in patients with MASH.^93^,^96^ APASL have suggested that in the presence of metabolic syndrome and the “cut-of” values of alcohol intake in MAFLD should be set lower than the apparent “threshold levels”.^2^ Consequently, individuals with MASLD should be advised to refrain from consuming alcohol, and if complete abstinence is not feasible, they should limit their intake to the smallest quantity possible.

HEPNET RECOMMENDATION# 12

12. Metabolic Dysfunction Associated Steatohepatitis (MASH) may be diagnosed if there is deranged liver enzymes and/or evidence of liver fibrosis in patients with MASLD.

We suggest to defined alcohol consumption based on daily intake as level I-III.

- ***Level-I*.** Less than 30gm/day for men and 20/day for women, this alcohol intake is presumed to be save as there is no evidence suggesting harmful effects even with existing liver disease.
- ***Level-II*.** More than Level-I but less than 60gm/day for men and 30gm/day for women, this amount of alcohol intake is considered safe but evidence suggesting that in patients with existing liver disease may be harmful and result in disease progression and clinical deterioration.
- ***Level-III*.** Alcohol intake equal to or more than 60gm/day for men and 30gm/day for women are harmful for liver even in absence of any existing liver disease.

HEPNET RECOMMENDATION# 13-16

13. Patients with Metabolic Dysfunction, as listed above, & HS with history of alcohol intake more than Level-I limits & with level-II limits may be diagnosed as MetALD.

14. Patients with Metabolic Dysfunction, as listed above, & HS with history of significant alcohol intake i.e. Level-III may be diagnosed as primary ALD with MD

15. Patients with HS with history of significant alcohol abuse disorder i.e. level-III without any of the metabolic risk factors may be classified as primary ALD without metabolic dysfunction (MD).

16. Those who neither have any MD nor any alcohol intake, but still have HS may be looked for DILI, Monogenic disorders, other rear causes or cryptogenic SLD.

### MetALD:

Patients with Metabolic Dysfunction, as listed above, & HS with history of alcohol intake more than Level-I limits as defined above & with Level-II limits may be diagnosed as MetALD.

HEPNET RECOMMENDATION# 17

17. All patients with advance fibrosis or cirrhosis should be put on hepatocellular carcinoma (HCC) surveillance, alpha-fetoprotein & US Liver every six months.

### ALD with MD:

Patients with Metabolic Dysfunction, as listed above, & HS with history of significant alcohol intake i.e. Level-III as defined above may be diagnosed as primary ALD with MD.

### ALD without MD:

Patients with HS with history of significant alcohol abuse disorder level-III without any of the metabolic risk factors listed above may be classified as primary ALD without metabolic dysfunction (MD).

### Drug induced liver injury (DILI):

Patients with HS with no history of alcohol abuse disorder & without any of the metabolic risk factors listed above, with history of the drugs causing HS may be classified as HS related to DILI.

### Monogenic Disorders:

SLD is a complex metabolic condition there are certain monogenic disorders that can also predispose individuals to this condition. Auto-brewery syndrome is a condition with endogenous ethanol production which can lead to HS and even cirrhosis. Some disorders of mitochondrial long-chain fatty acid oxidation and the carnitine shuttle impair the body’s ability to metabolize them and result in their accumulation in the liver and the development of SLD. While lysosomal storage diseases, peroxisomal disorders, and certain genetic forms of metabolic syndrome, have also been associated with the development of HS.

### Others:

There are some other miscellaneous conditions also associated with HS such as acute fatty liver of pregnancy, HELLP syndrome HCV GT3, Wilson disease, lipodystrophy, starvation, parental nutrition and elevated lipoproteinemia. These all can present as HS on Ultrasound.

### Cryptogenic:

When none of the above causes are present still HS may be cryptogenic.

## HEPATOCELLULAR CARCINOMA (HCC) AND MASLD

HCC is the primary liver tumor and have been seen in patients with advance fibrosis and cirrhosis due to many causes.^38^,^39^ Studies from across the globe suggest that NAFLD/MASLD is now ranked one of the most frequent causes of HCC.^35^,^41^,^97^-^99^ Liver fibrosis and advance cirrhosis are well established risk factors for development of HCC.^38^,^39^ Compared to other HCC patients, people with MASLD-related HCC are older, have a shorter survival time, are more likely to have cardiac disease due to metabolic risk factors in addition to liver fibrosis and cirrhosis, and are more likely to pass away from their original liver cancer.^35^,^39^,^41^,^98^-^100^ Surveillance tools for HCC based on international guidelines include contrast-enhanced ultrasound (CEUS), biannual abdominal ultrasound with serum AFP, and dynamic contrast MRI.^101^-^104^ The ultrasound and ALP when combined is the most cost-effective strategy for early HCC detection in real world setting, with a sensitivity of 90% and specificity of 83%.^102^

## HEPNET ALGORITHM FOR SCREENING & DIAGNOSIS OF SLD.

**Fig.2:**
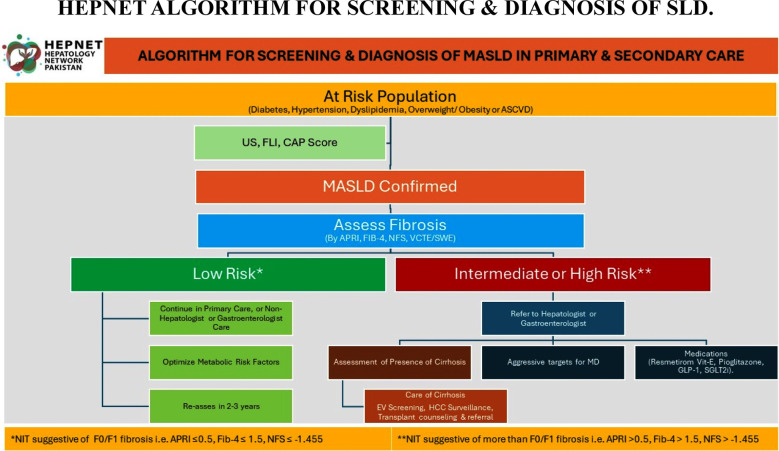
Diagnosis of MASLD.

**Table:** KEY RECOMMENDATIONS.

S.No	Key Recommendation	Strength
1	HEPNET recommends nomenclature of Metabolic Dysfunction Associated Steatotic Liver Disease (MASLD) should be adopted to replace existing term NAFLD in Pakistan	I
2	Diagnosis of Steatotic Liver Disease (SLD) should be established based on any imaging evidence of HS (ultrasound, transient elastography, CT scan or MRI).	I
3	All patients with SLD on imaging should undergo focused clinical and laboratory evaluations for metabolic risk factors, alcohol intake (AUDIT-C or CAGE questioner), liver biochemistry & viral serology.	I
4	NIT can be used in patients with SLD to diagnose, evaluate its severity & monitor the disease progression.	II
5	FLI is recommended to confirm, quantify & monitor HS.	II
6	APRI, FIB-4 or NFS are recommended to confirm, quantify & monitor liver fibrosis.	I
7	Fibro scan & SWE are recommended to confirm, quantify & monitor liver fibrosis.	I
8	Controlled attenuated parameters measured by either VCTE or SWE are effective in diagnosing SLD, quantify & monitor its progression.	I
9	Liver Biopsy should only be restricted in therapeutic drug trails or where alternate diagnosis is being sought.	I
10	MASLD should be diagnosed based on if there are any single associated metabolic risk factors is present namely Pre-diabetes, Diabetes, Hypertension, Obesity (define by Asian cut-offs of BMI or Central Obesity) or Dyslipidemia along with imaging evidence HS.	I
11	Insulin resistance (HOMA-IR) should be checked in patients without diabetes	II
12	Metabolic Dysfunction Associated Steatohepatitis (MASH) may be diagnosed if there is deranged liver enzymes and/or evidence of liver fibrosis in patients with MASLD.	I
13	Patients with Metabolic Dysfunction, as listed above, & HS with history of alcohol intake more than level-I limits & with level-II limits may be diagnosed as MetALD.	II
14	Patients with Metabolic Dysfunction, as listed above, & HS with history of significant alcohol intake i.e. Level-III may be diagnosed as primary ALD with MD	I
15	Patients with HS with history of significant alcohol abuse disorder i.e. level-III without any of the metabolic risk factors may be classified as primary ALD without metabolic dysfunction (MD).	I
16	Those who neither have any MD nor any alcohol intake, but still have HS may be looked for DILI, Monogenic disorders, other rear causes or cryptogenic SLD.	I
17	All patients with advance fibrosis or cirrhosis should be put on hepatocellular carcinoma (HCC) surveillance, alpha-fetoprotein & US Liver every six months.	I

## CONCLUSION

In the current epidemic of twin diabetes & obesity with ever increasing incidence of liver dysfunction the previous terminology of NAFLD was not only missing main drivers of the disease pathophysiologically but it was also stigmatic with terms like “fatty” & “alcoholic”. It also needed to exclude alcohol intake altogether leaving many patients without diagnosis as there was significant no patients with dual pathology. More so with previous terminology being diagnosis of exclusion and needing biopsy for confirmation remained major obstacle for labeling the disease. Now with overarching term like Steatotic Liver Disease (SLD) and positive and simplified algorithmic approach to diagnosis and classification it will be more easier for physicians to diagnose it and classify it.

### Authors Contributions:

**JIF:** Conceptualized the paper and assigned different segments to authors to review data. **AA**: Reviewed the draft for initial write-up. **ABN:** Wrote initial draft of introduction and did data search and reviewed the data. **ZA:** Wrote final draft, reviewed all citations, made figures and charts. **BFZ:** Drafted statements and got consensus over these by all authors. **AAN:** Reviewed the data and approved the draft of statements. **SM:** Reviewed the data and approved the draft of statements. **ZYH:** Drafted the diagnosis segment and reviewed the relevant data. **AAC:** Drafted the segment of non-invasive tests. **AS:** Wrote initial draft and searched all relevant data. **BA:** Wrote initial draft and searched all relevant data. **LK:** Wrote first submission. **MS:** Reviewed the data and approved the draft of statements. **AAK:** Reviewed the data and approved the draft of statements. **SMZA:** Wrote initial draft and searched all relevant data. **ZA:** Reviewed & corrected the final draft. **MS:** Approved the draft of position statements. All authors voted for final drafted statements and approved them.
